# Metabolism of the Pacific oyster, *Crassostrea gigas*, is influenced by salinity and modulates survival to the Ostreid herpesvirus OsHV-1

**DOI:** 10.1242/bio.028134

**Published:** 2018-02-15

**Authors:** Marine Fuhrmann, Lizenn Delisle, Bruno Petton, Charlotte Corporeau, Fabrice Pernet

**Affiliations:** 1 Ifremer/LEMAR UMR 6539 (UBO/CNRS/IRD/Ifremer), Technopole de Brest-Iroise, 29280 Plouzané, France; 2 Ifremer/LEMAR UMR 6539 (UBO/CNRS/IRD/Ifremer), Presqu’île du vivier, 29840 Argenton, France

**Keywords:** Bivalve, Disease, Environment, Metabolism, Mortality risk, Salinity

## Abstract

The Pacific oyster, *Crassostrea gigas*, is an osmoconforming bivalve exposed to wide salinity fluctuations. The physiological mechanisms used by oysters to cope with salinity stress are energy demanding and may impair other processes, such as defense against pathogens. This oyster species has been experiencing recurrent mortality events caused by the Ostreid herpesvirus 1 (OsHV-1). The objectives of this study were to investigate the effect of salinity (10, 15, 25 and 35‰) on energetic reserves, key enzyme activities and membrane fatty acids, and to identify the metabolic risk factors related to OsHV-1-induced mortality of oysters. Acclimation to low salinity led to increased water content, protein level, and energetic reserves (carbohydrates and triglycerides) of oysters. The latter was consistent with lower activity of hexokinase, the first enzyme involved in glycolysis, up-regulation of AMP-activated protein kinase, a major regulator of cellular energy metabolism, and lower activity of catalase, an antioxidant enzyme involved in management of reactive oxygen species. Acclimation to salinity also involved a major remodeling of membrane fatty acids. Particularly, 20:4*n*-6 decreased linearly with decreasing salinity, likely reflecting its mobilization for prostaglandin synthesis in oysters. The survival of oysters exposed to OsHV-1 varied from 43% to 96% according to salinity (Fuhrmann et al., 2016). Risk analyses showed that activity of superoxide dismutase and levels of proteins, carbohydrates, and triglycerides were associated with a reduced risk of death. Therefore, animals with a higher antioxidant activity and a better physiological condition seemed less susceptible to OsHV-1.

## Abstract (precis)

**Summary:** Environmental salinity influences energetic reserves, enzyme activity, membrane fatty acids of the Pacific oyster and modulates survival to viral infection.

## INTRODUCTION

Oysters live in estuaries and bays where they are exposed to wide salinity fluctuations. Like most marine invertebrates, oysters are osmoconformers. They have an osmotic concentration in their body fluids equal to that of the surrounding seawater; however, they maintain concentrations of salts that are out of equilibrium with the environment, and this requires extensive regulation (Hochachka and Somero, 2002). Therefore, oysters allocate energy to maintain cell volume and integrity (Carregosa et al., 2014a; Dickinson et al., 2012; Gilles, 1972; Neufeld and Wright, 1996; Shumway, 1977a,b; Shumway et al., 1977). The increasing energetic demand during an exposure to salinity change may divert resources from other processes, such as defense against pathogens.

Salinity changes influence disease risk in oysters, directly by acting on the pathogen, the host or both (Brothers et al., 2000; Burge et al., 2014; Reid et al., 2003; Soudant et al., 2013). In a companion paper, we showed that the survival of oysters exposed to the Ostreid herpesvirus (OsHV-1) and acclimated at 10‰ was higher (95.8%) than that of oysters held at 15, 25 and 35‰, where survival was 73.2, 43.2 and 61.9%, respectively (Fuhrmann et al., 2016). The infectivity of OsHV-1, as revealed by the level of OsHV-1 DNA and RNA in oyster, was low at 10‰, but it was similar at 15, 25 and 35‰. Therefore, the differences in survival among salinities between 15‰ and 35‰ could reflect changes in the metabolic response of the host to salinity rather than OsHV-1 infection.

The energetic budget of oysters is altered when subjected to environmental stressors (Sokolova et al., 2012) and disease (Pernet et al., 2012, 2014). Although carbohydrates and lipids are not osmotically important, they may play important roles in supplying energy requirement during osmotic stress in osmoconformers (Ballantyne and Moyes, 1987). Also, these energetic reserves play a role in the outcome (death or recovery) of the infection by OsHV-1 (Pernet et al., 2012, 2014). In order to better understand the physiological mechanisms that may play a role in the survival of oysters to OsHV-1 and/or salinity exposure (Fuhrmann et al., 2016), we focused on a suite of metabolic parameters that are described below.

The AMP-activated protein kinase (AMPK) is a major regulator of cellular energy metabolism by activating ATP production pathways and blocking ATP consumption (Jeon, 2016). Expression of AMPK is modulated by salinity in marine shrimp and finfish species (Zeng et al., 2016) and it regulates autophagy (Jeon, 2016), a conserved intracellular pathway playing a key role in innate immunity which protects oysters from OsHV-1 infection (Moreau et al., 2015).

In marine bivalves, carbohydrates constitute a major energy reserve (Berthelin et al., 2000; Gabbot, 1975) that can be used for fueling defense against pathogens (Plana et al., 1996). Carbohydrates are converted to pyruvate by means of glycolysis. The first step in glycolysis is the phosphorylation of glucose by hexokinase (HK). Activity of HK declines in the Eastern oysters, *Crassostrea virginica*, at low salinity to achieve homeostasis of metabolic function (Ballantyne and Berges, 1991). Also, activity of HK is lower in oysters infected by OsHV-1 than in uninfected oysters (Pernet et al., 2012; Tamayo et al., 2014).

The enzyme citrate synthase (CS) is a pacemaking enzyme in the first step of the citric acid cycle, consisting of a series of chemical reactions to generate energy through the oxidation of acetyl-CoA derived from carbohydrates, lipids and proteins. Activity of CS correlates with respiration rate in marine invertebrates and can be used as indicator of physiological condition (Dahlhoff et al., 2002). Although salinity (Ballantyne and Berges, 1991) or infection by OsHV-1 (Pernet et al., 2012; Tamayo et al., 2014) do not influence CS activity in oyster, the interaction of salinity and OsHV-1 on CS has never been investigated.

The main cellular defense mechanism in bivalves relies on phagocytosis by haemocytes. This process ends by the internal destruction of pathogens through an increased production of reactive oxygen species (ROS) that are toxic to invaders (Bachère et al., 1991; Torreilles et al., 1996). Accumulation of ROS can result in oxidative stress in the host (Lesser, 2006) which is normally prevented by several enzymes (Hermes-Lima, 2004; Sies, 1993). Studies have highlighted the importance of superoxide dismutase (SOD) and catalase (CAT) during acclimation to salinity (An and Choi, 2010; Carregosa et al., 2014b) and exposure to pathogen in bivalves (Genard et al., 2013; Richard et al., 2016).

Membrane lipid composition is altered to enable physiological adaptation of organisms to their physical environment (Hazel and Williams, 1990; Hochachka and Somero, 2002). The best example of this is the remodeling membrane lipids by ectothermic animals, including bivalves, to counteract the effect of temperature on membrane fluidity (Hall et al., 2002; Hazel, 1995; Pernet et al., 2007). In addition to the physical factors of temperature, the chemical composition of the aqueous phase in contact with a membrane may also exert a profound influence on membrane structure and function (Hazel and Williams, 1990). The only study related to a marine bivalve shows that salinity modulates the fatty acid composition of gill mitochondrial membranes of the oyster *C. virginica* (Glémet and Ballantyne, 1995).

In addition, 20 carbon polyunsaturated fatty acids (PUFAs) represent precursors of eicosanoids, a group of hormones involved in inflammatory processes and stress response (Smith and Murphy, 2003). Among the many functions of eicosanoids in invertebrates, they are involved in salt and water transport physiology and cellular immune response (Stanley-Samuelson, 1994). Membrane lipids of bivalve haemocytes are characterized by elevated amounts of arachidonic acid (20:4*n*-6), presumably to regulate immune responses (Delaporte et al., 2003).

The objectives of this study were (i) to investigate the effect of salinity (10, 15, 25 and 35‰) and infection (exposed to OsHV-1 versus unexposed) on the metabolic response of the oyster *C. gigas*, and (ii) to identify metabolic risk factors related to disease-induced mortality. Energetic reserves, key enzyme activities and the composition of fatty acid from polar lipids may change among salinities and likely modulate the host response to pathogens and its survival.

## RESULTS

### Effect of salinity and infection on the metabolic response of oysters

#### Proximate composition

**Fig. 1.**
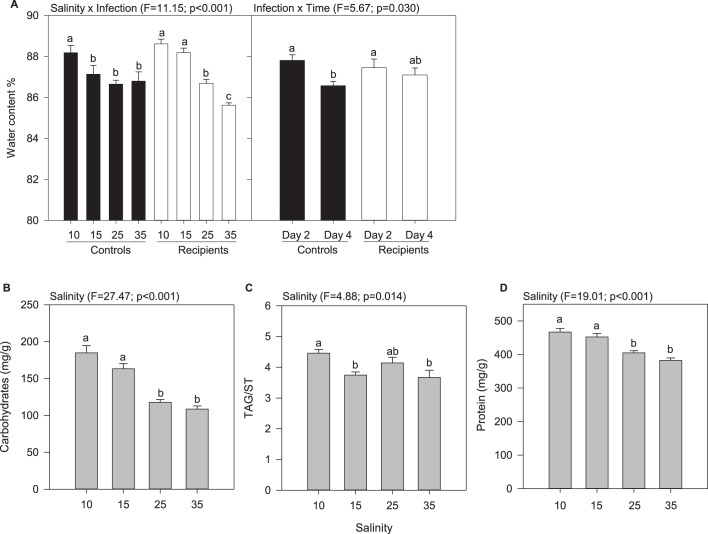
**Proximate composition of oyster tissues as a function of salinity, infection, and time.** Only significant effects are represented. Data are means± s.e.m. (*n*=3 tanks). Letters indicate significant differences. (A) Water content as a function of salinity × infection and infection × time. (B) Carbohydrate content as a function of salinity. (C) Level of triglycerides relative to sterols (TAG/ST) as a function of salinity. (D) Protein content as a function of salinity.

The water content of oyster tissues varied as a function of salinity×infection and infection×time. This parameter decreased with increasing salinity (Fig. 1A). Significant differences were observed between controls at 10‰ that is it and controls at higher salinities, and between recipients at 10-15‰ and recipients at 25-35‰ (Fig. 1A; Tables S1 and S2). Also, the water content of oyster tissues slightly decreased with time in controls (Fig. 1A).

The carbohydrate content in oysters decreased with increasing salinity. In oysters held at 10-15‰, it was 54% higher than that of animals maintained at 25-35‰ (Fig. 1B; Tables S1 and S2). The level of triglycerides relative to sterols (TAG/ST) in oysters at 10‰ was ∼20% higher than in oysters held at higher salinities, except at 25‰ where difference was not significant (Fig. 1C; Tables S1 and S2). The protein content of oyster tissues at 10-15‰ was 17% higher than in animals at 25-35‰ (Fig. 1D; Tables S1 and S2).

#### Energetic- and antioxidant-related enzymes

**Fig. 2.**
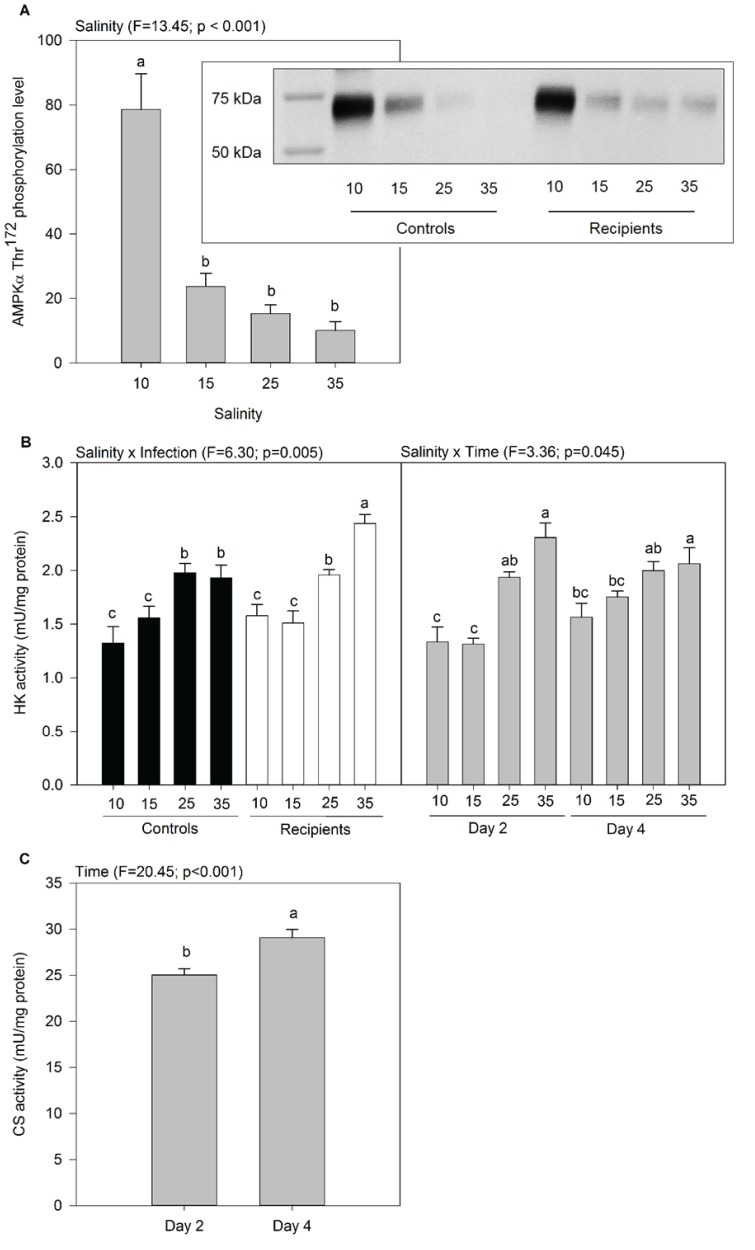
**Activities of enzymes related to energetic metabolism in oyster tissues as a function of salinity, infection, and time.** Only significant effects are represented. Data are means±s.e.m. (*n*=3 tanks). Letters indicate significant differences. (A) Quantification of Thr172 phosphorylation of AMPKα as a function of salinity. The protein values presented on the graph were quantified on three western blots and expressed in relative levels of OD/mm^2^. Inset is a representative image of western blot of AMPKα Thr172 phosphorylation (62 kDa) in whole oyster tissues protein extract according to salinity, in controls and recipients. (B) Activity of hexokinase (HK) as a function of salinity × infection and salinity × time. (C) Activity of citrate synthase (CS) as a function of time.

The level of AMPK measured in oyster at day 4 was higher at 10‰ than at other salinities (Fig. 2A). The activity of HK varied as a function of salinity×infection and salinity×time. Activity of HK generally increased with increasing salinity in both controls and recipients, and was the highest in infected oysters at 35‰ (Fig. 2B). At days 2 and 4 in oysters held at 25-35‰, the HK activity was higher than at 10-15‰ at day 2 (Fig. 2B; Tables S3 and S4). The activity of CS in oyster tissues was not influenced by salinity or infection, but it slightly increased between day 2 and 4 (Fig. 2C; Tables S3 and S4).

**Fig. 3.**
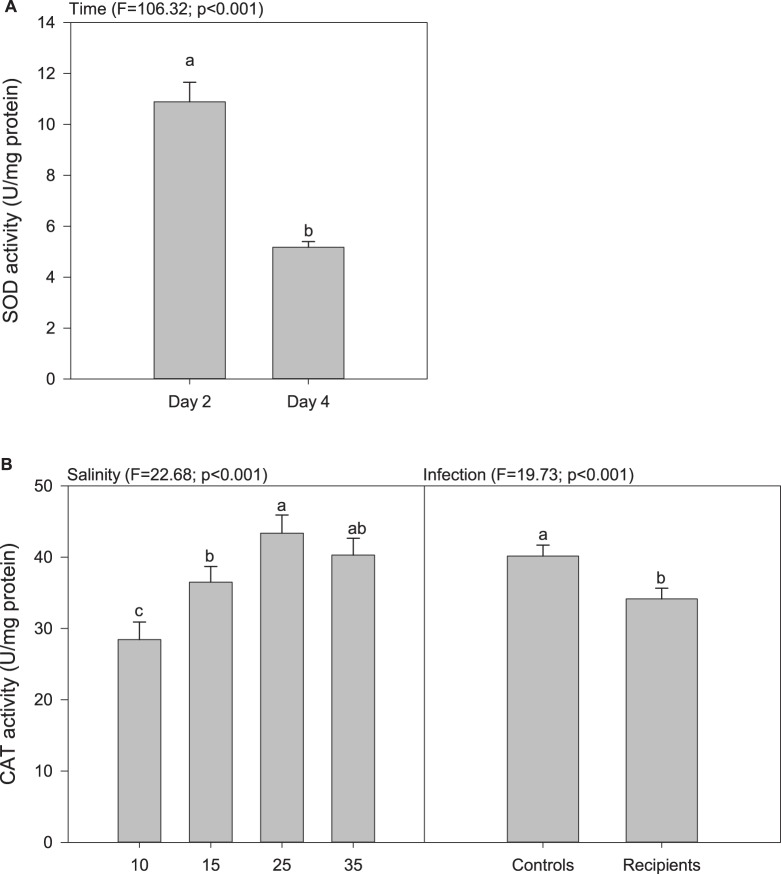
**Activities of antioxidant related enzymes in oyster tissues as a function of salinity, infection, and time.** Only significant effects are represented. Data are means±s.e.m. (*n*=3 tanks). Letters indicate significant differences. (A) Activity of superoxide dismutase as a function of time. (B) Activity of catalase as a function of salinity and infection.

The activity of SOD in oyster tissues decreased markedly between days 2 and 4, irrespective of salinity and infection (Fig. 3A; Tables S3 and S4). The activity of CAT increased with increasing salinity up until reaching a plateau at 25‰, and it was 15% lower in recipients than in controls (Fig. 3B; Tables S3 and S4).

#### Fatty acids remodeling of polar lipids

The fatty acid composition of the polar lipids of oysters at day 4 was influenced mainly by salinity (Table 1). The unsaturation index (UI) was slightly lower in animals held at 10‰ (mean UI=264) compared to that of individuals maintained at 15 and 25‰ (mean UI=270), whereas the UI of oysters held at 35% was intermediate.

**Table 1.**
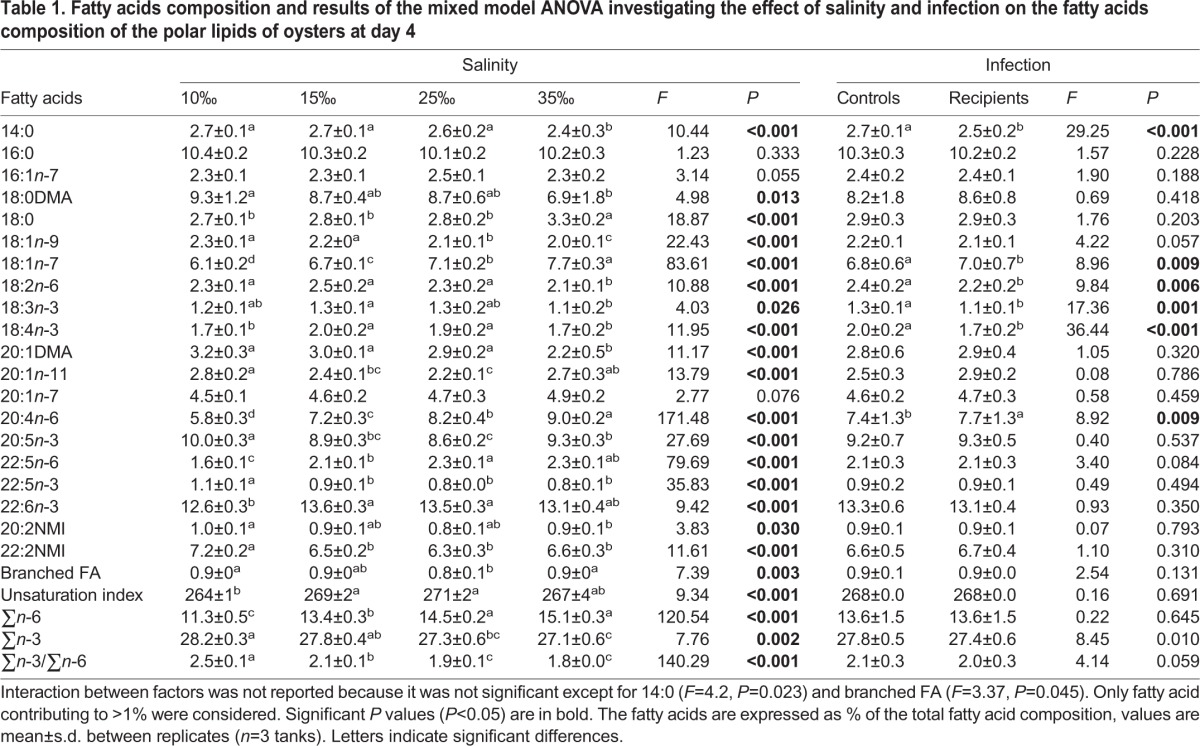
**Fatty acids composition and results of the mixed model ANOVA investigating the effect of salinity and infection on the fatty acids composition of the polar lipids of oysters at day 4**

The levels of dimethyl acetals (DMA; 18:0 and 20:1), 18:1*n*-9 and 22:5*n*-3 decreased with increasing salinity; whereas the levels of 18:0, 18:1*n*-7 and 20:4*n*-6 increased with increasing salinity (Table 1; Table S5). The fatty acid 20:4*n*-6 showed the strongest correlation with salinity (% 20:4*n*-6=0.12×salinity+4.98, r^2^=0.94, *P*<0.001), and it increased from 5.8% to 9.0% when salinity rose from 10‰ to 35‰. Also, the level of 20:4*n*-6 in controls was 5% lower than that of recipients. The overall level of *n*-6 fatty acids (FA) increased with increasing salinity; whereas the opposite relationship was observed for the *n*-3 FA (Table 1; Table S5). Therefore, the ∑*n*-3/∑*n*-6 decreased from 2.5 to 1.8 with increasing salinities from 10‰ to 35‰ (Table 1). The levels of non-methylene interrupted fatty acids (NMI) and 20:1*n*-11 dropped with an increase in salinity from 10‰ up to 15-25‰ and increased thereafter at 35‰.

### Metabolic risk factors related to disease-induced mortality

The risk model adjusted for the effect of salinity revealed that activity of SOD at day 4 showed the strongest association with mortality risk (Table 2). Each increase of 1 unit of activity of SOD decreased the hazard of mortality by 12% (Table 2). Otherwise, the overall physiological condition of oysters was associated with mortality risk. For instance, decreasing water content and increasing protein, carbohydrate and triglyceride levels were associated with a lower risk of death. Also, increasing the activity of CS at day 2 was associated with a higher risk of mortality, but it was the opposite at day 4 when the first mortalities were observed. Finally, increasing levels of 20:1*n*-11 and 22:5*n*-3 were associated with higher risk of mortality (Table 2). Only the most significant explanatory covariate (SOD activity at day 4) was included in the multiple regression model.

**Table 2.**
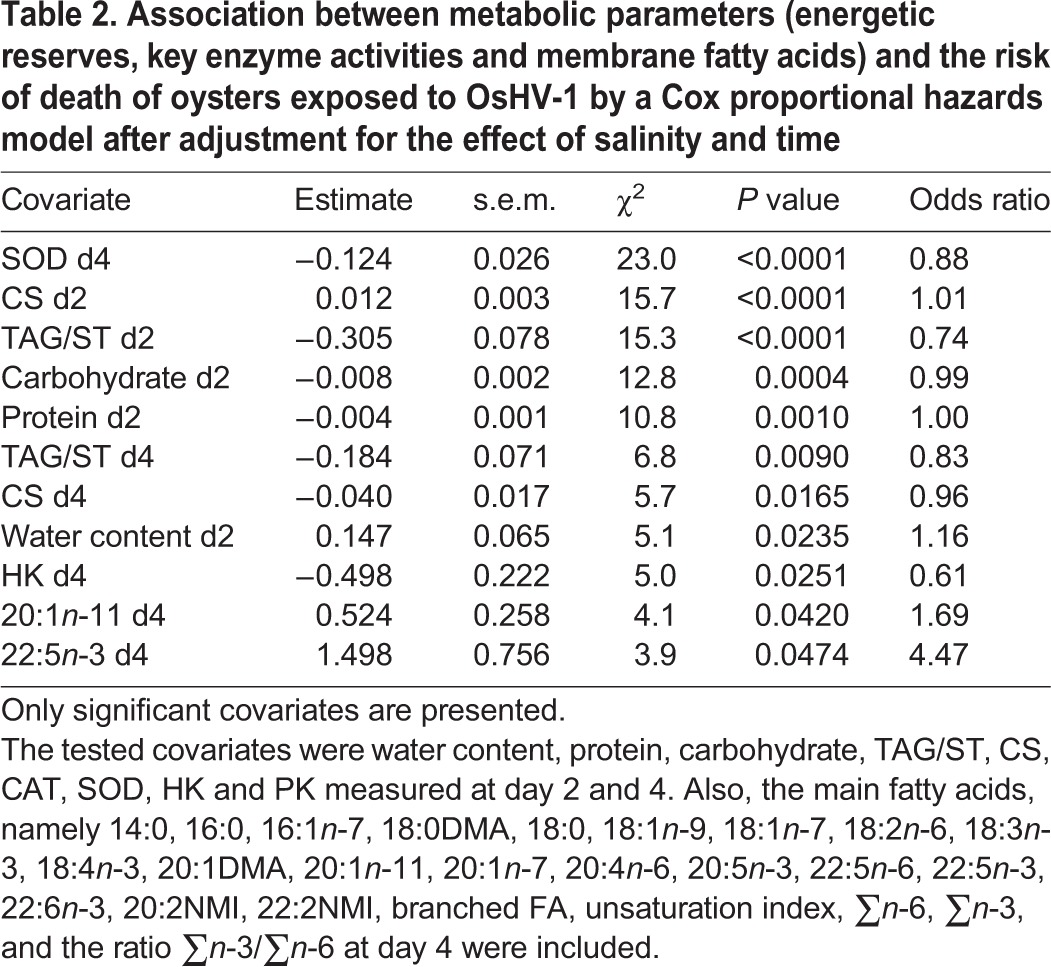
**Association between metabolic parameters (energetic reserves, key enzyme activities and membrane fatty acids) and the risk of death of oysters exposed to OsHV-1 by a Cox proportional hazards model after adjustment for the effect of salinity and time**

Univariate association between metabolic parameters and the risk of death of oysters exposed to the SI without adjustment for salinity showed that the level of ∑*n*-3/∑*n*-6 was strongly associated with mortality risk (Table S6). Most *n*-3 PUFAs (22:5*n*-3, ∑*n*-3, 20:5*n*-3), and the fatty acids related to plasmalogenic phospholipids (NMI, DMA and 20:1*n*-11), were associated with a lower risk of death, whereas the opposite was observed for *n*-6 PUFAs (∑*n*-6, 22:5*n*-6 and 20:4*n*-6). Also, the relative water content, the total amount of proteins and carbohydrates and AMPK were associated with a lower risk of mortality, whereas this was the inverse for the activity of HK, an enzyme involved in the first step of glycolysis. Finally, the antioxidant activity of CAT, the level of 22:6*n*-3 and the insaturation index of the fatty acids in the polar lipids were associated with a higher risk of death in oysters. These associations were confounded with the effect of salinity.

## DISCUSSION

### Effect of salinity on the metabolic response of oysters

#### Energetic reserves

The water content in oyster tissues had an increase of 3% with decreasing salinity from 35‰ to 10‰. This result is consistent with the fact that when salinity decreases, the extracellular concentration is lowered and cells of osmoconformers takes up water. In *C. gigas*, the water content increases from 75 to 84% when seawater is diluted by 70% (Shumway, 1977a). Overall, these changes in hydration of tissues are relatively small compared to those occurring in the surrounding seawater. In the absence of any regulating mechanism, an animal exposed to 50% diluted seawater would expect to double its cellular water content (Shumway, 1977a). Therefore, oysters actively regulate their internal fluid concentrations in response to salinity to maintain their cell volume relatively constant.

In our study, the protein concentrations in oysters increased with decreasing salinity. Protein synthesis may have occurred in response to low salinity to reduce the concentration of amino acid in the cell, so that the cell remains isotonic with the surrounding seawater. Intracellular concentration of free amino acids is an important parameter for controlling cell volume in response to changes in salinity in osmoconformers (Hawkins and Hilbish, 1992; Shumway et al., 1977; Yancey et al., 1982). Such compensatory mechanisms allow acclimation to salinity but they can affect energy status (Sokolova et al., 2011).

Carbohydrate levels in oysters held at 10-15‰ were higher than that of animals at 25-35‰. Consistently, activity of HK, the first enzyme involved in the glycolysis pathways, decreased with decreasing salinity. Similarly, HK activity in the oyster *C. virginica* decreased by a factor two when seawater is diluted by two-thirds (Ballantyne and Berges, 1991).

Furthermore, TAG level (relative to ST) was the highest in oysters at 10‰. Accordingly, the activity of 3-hydroxyacyl CoA dehydrogenase, an enzyme involved in the beta-oxidation, the catabolic process of fatty acids, is reduced in oyster *C. virginica* at low salinity (Ballantyne and Berges, 1991).

In our study, energetic reserves (carbohydrates and triglycerides) were higher in oysters maintained at low salinity for 14 to 16 days (2 to 4 days post-infection), and this is consistent with HK activity which showed the lowest activity at 10 and 15‰. In contrast, the lipid content of oyster *C. virginica* maintained at 15‰ for 11 weeks was 50% lower than that of animals acclimated at 30‰ (Dickinson et al., 2012). Discrepancies between studies may reveal that the short-term effects of exposure to low salinity on the energetic metabolism are different from those observed on the long-term.

More broadly, the higher level of energetic reserves in oysters at low salinity may reflect that when the surrounding salinity is changed, they close their valves and their oxygen consumption rate decreases (Gosling, 2003; Kim et al., 2001; Shumway, 1977a). This protective strategy against osmotic stress is in favor of conservation of energy reserves by reducing respiration (Kim et al., 2001). In our study, growth rate of oysters at 10‰ was null during the first 11 days of acclimation, and growth ranked as 10‰<15‰<25‰=35‰ (Fuhrmann et al., 2016). Also, filtration rates of oysters at 10 and 15‰ were initially lower than those of oysters at 25-35‰. Filtration rates then increased during the first 2 to 6 days of acclimation until reaching those of oysters at 25-35‰. The reduction in filtration and growth of oysters at low salinity are in agreement with energy use reduction.

Concomitantly, AMPK was up-regulated at low salinity, likely reflecting an excess in the energy demand during salinity stress, as reported in the whiteleg shrimp *Litopenaeus* *vannamei* (Xu et al., 2016). However, AMPK generally stimulates glycolysis by up-regulating HK activity, which is not the case in our study. Low salinity may have inhibited HK independently of AMPK, as reported in oysters exposed to trace levels of pesticides (Epelboin et al., 2015).

#### Antioxidant activities

Although activity of SOD in oysters remained unaffected by salinity, activity of CAT decreased with decreasing salinity. In contrast, activities of SOD and CAT in gills and digestive gland of the ark shell *Scapharca broughtonii* and in whole-tissues of clams *Ruditapes* spp. increase in response to low salinity (An and Choi, 2010; Carregosa et al., 2014b). Discrepancies among studies may reflect differences in duration of acclimation to salinity and species. In our study, oysters were acclimated for 13 to 15 days before sampling, whereas ark shells and clams were exposed to salinity change for 5-6 days (An and Choi, 2010; Carregosa et al., 2014b). The specific time response of antioxidant enzymes during acclimation to salinity needs further investigation.

The production of ROS can lead to peroxidation of lipid membranes (Abele and Puntarulo, 2004; Sies, 1993; Storey, 1996), as observed in ark shell and clams at low salinity (An and Choi, 2010; Carregosa et al., 2014b). In our study, the UI of membrane lipids, also viewed as a peroxidation index (Hulbert, 2003), was slightly lower in animals held at 10‰ (−2.2%) compared to that of individuals maintained at 15 and 25‰, whereas the UI of oysters held at 35% was intermediate. These variations in UI are small (±2%) compared to those that can occur in response to a change of temperature in bivalves (∼20% when ΔT°C=20°C; Pernet et al., 2007). Therefore, there was no evidence of membrane lipid peroxidation in response to salinity.

The levels of dimethyl acetals (18:0 DMA and 20:1 DMA) decreased with increasing salinity. These DMA are associated with plasmalogen in bivalves (Kraffe et al., 2004) and they act as endogenous membrane antioxidants (Hulbert et al., 2014). Higher levels of DMA in oysters at low salinity may have protected them from ROS and compensated for the reduced activity of CAT at 10-15‰.

#### Fatty acids remodeling of polar lipids

The ∑*n*-3/∑*n*-6 ratio in the polar lipids decreased markedly with increasing salinities in whole tissues of oysters, and this trend mainly reflects the positive correlation between salinity and 20:4*n*-6. The only study that investigated the effect of salinity on the membrane fatty composition of bivalves demonstrated that the remodeling of fatty acids is specific to lipid classes (Glémet and Ballantyne, 1995). For instance, the ∑*n*-3/∑*n*-6 ratio and the level of 20:4*n*-6 in gill mitochondria of the oyster *C. virginica* are higher at higher salinity in phosphatidylinositol, but not in the other phospholipid classes. Although the current study differed from Glémet and Ballantyne (1995) in several respects, i.e. species, tissues and lipid classes were different, making comparisons difficult, they both demonstrated a major fatty acid remodeling of the polar lipids in response to salinity, and this may have consequences on membrane fluidity, permeability and on activities of ion pumps and membrane-bound enzymes (Hulbert, 2003). It is, however, noteworthy that the fatty acid remodeling in response to salinity did not involve a change in unsaturation index of membrane fatty acid as generally observed during thermal acclimation and adaptation (Hall et al., 2002; Hazel, 1995; Pernet et al., 2007).

The level of 20:4*n*-6 in oysters increased linearly with salinity. This C20 fatty acid is a precursor of prostaglandins and related eicosanoids in eukaryotic cells (Smith and Murphy, 2003). Among the many functions of eicosanoids, they are involved in salt and water movements across epithelial tissues and lead to release of osmotically active species and restoration of normal cell volume (Hochachka and Somero, 2002; Stanley-Samuelson, 1994). For instance, hyposmotic stress increases the synthesis and release of prostaglandins in gills of the mussel *Modiolus demissus*, and this effect is exacerbated by the addition of 20:4*n*-6 (Freas and Grollman, 1980). Also, injection of 20:4*n*-6 modulate sodium transport in freshwater mussels (Graves and Dietz, 1982). Finally, there is a positive correlation between 20:4*n*-6 and prostaglandin E metabolite in *C. gigas* (Seguineau et al., 2011). Therefore, the reduction of 20:4*n*-6 in oyster tissues at low salinity may be related with its mobilization for prostaglandin synthesis and play a role in acclimation to salinity.

### Metabolic risk factors related to disease-induced mortality

Risk analyses showed that higher protein, carbohydrate, and triglyceride levels and lower water content were associated with a lower risk of death, suggesting that animals with a better physiological condition were more likely to amount a response to OsHV-1. This agrees with previous studies where oysters with high levels of carbohydrates and triglycerides are more resistant or tolerant to OsHV-1 (Pernet et al., 2012, 2014). Immune response may be energetically expensive and higher energetic reserves may lead to a stronger immune response which decreases the risk of death. It is possible that the energy sparing in low-salinity oysters allows fostering the immune response.

The CS activity in oysters was similar among salinities as previously observed in *C. virginica* (Ballantyne and Berges, 1991). Increased activity of CS before the onset of mortality (day 2) was associated with a higher risk of mortality in oysters infected with OsHV-1. This suggests that oysters that have a higher aerobic metabolism are less able to fight the virus. However, the opposite association was observed at day 4 when the first mortalities were observed. Therefore, there was a temporal shift in the effect of aerobic metabolism on disease susceptibility of oysters.

The activity of SOD measured at day 4 was associated with a reduced risk of mortality of oysters infected with OsHV-1. This effect was independent from that of environmental salinity which had no effect on the activity of SOD. This association may reflect that animals with a higher basal antioxidant activity with advanced OsHV-1 infection are less susceptible to the disease. Although changes in activity or gene expression of antioxidant enzymes has been reported during exposure of bivalves to pathogenic bacteria (Canesi et al., 2010; Genard et al., 2013; Labreuche et al., 2006; Richard et al., 2016), this phenomenon has never been related to viral infection. The expression of extracellular SOD was significantly higher in oysters selected for resistance (as compared to susceptible oysters) to summer mortalities, a phenomenon in which OsHV-1 was presumably involved (Fleury and Huvet, 2012).

The fatty acid and DMA composition of oysters exposed to OsHV-1 was associated with the risk of mortality, but this relationship was confounded with salinity so that it is impossible to establish causal relations. However, we found that the level of 20:4*n*-6 was higher in infected oysters than in controls. It is therefore likely that membrane fatty acids, and particularly the relative level of 20:4*n*-6, influence the immune response of oysters and their survival. It is now clearly established that membrane fatty acid composition of immune cells influences the immune response of higher animals (Calder, 2008), but also bivalves (Delaporte et al., 2007, 2006, 2003). In oysters, 20:4*n*-6 enhances immune parameters of haemocytes, whereas 20:5*n*-3 has the opposite effect (Delaporte et al., 2007, 2006).

This study showed that metabolism of the Pacific oyster is influenced by salinity and modulates survival to OsHV-1. Low salinity increased water content, protein level, energetic reserves of oysters, changed membrane fatty acids, reduced activity of hexokinase, up-regulated AMPK, and decreased activity of catalase. Activity of superoxide dismutase and levels of proteins, carbohydrates, and triglycerides were associated with a reduced risk of death caused by OsHV-1. Therefore, animals with a higher antioxidant activity and a better physiological condition seemed less susceptible to the virus. This highlights that environment strongly influences host metabolism and the outcome of pathogen infection.

## MATERIALS AND METHODS

### Experimental design

Details about the rearing procedure and experimental design are presented in Fuhrmann et al. (2016). Oysters were produced in hatchery at the Ifremer marine Station (Argenton, France) according to Petton et al. (2015). At the onset of the experiment (17 July 2014) oysters were 4 months old, 0.8±0.2 g wet weight and 19.5±2.5 mm shell length (mean±s.d.). They were maintained at 21.1°C, 35.2‰ salinity, and fed with *Chaetoceros muelleri* (CCAP 1010/3) and *Tisochrysis lutea* (CCAP 927/14) (1:1 in dry weight). Phytoplankton concentration was maintained at *ca.* 1500 μm^3^ μl^−1^ algae. A light bubbling was added to each tank to maintain the oxygen level between 85 and 100% of saturation.

From 17 to 28 July 2014, a subsample of the oyster cohort was placed at four salinities (10.2±0.4, 15±0.8, 24.9±0.4, 35.4±0.2‰) in 45-liter tanks (*n*=6 tanks per salinity) in a flow-through system. The salinities were obtained by melting 1 µm filtered and UV sterilized seawater (35‰) with freshwater. Each tank contained 310 oysters (total biomass=250 g). These oysters were then used as pathogen recipients or controls.

The remaining oysters were transferred to a farming area of the Bay of Brest (Brittany, France, 48° 20′ 06.19″ N, 4° 19′ 06.37″ W) during a disease outbreak caused by OsHV-1 on 22 July 2014. After 6 days of field exposure, few individuals died. Alive oysters were moved back to the laboratory. The DNA of OsHV-1 in these oysters was quantified according to the procedure described in Fuhrmann et al. (2016). These oysters were positive for OsHV-1 [the level of virus DNA was 8.2×10^8^±5.5×10^8^ copies mg^−1^ fresh weight (mean±s.d.), *n*=3 pools of 15 oysters], and they were used as pathogen donors.

On 28 July 2014 (day 0), the density of acclimated oysters was standardized at *ca.* 160 g in each tank, to remedy differences in growth rates among salinity treatments. In the meantime, pathogen donors were placed in two 45-liter flow-through tanks connected to each other following a cascading system, and filled with seawater at 35‰ at 21°C. The seawater surrounding the donors in the last tank was used as source of infection (SI). The SI was connected to half of tanks which contained the recipient oysters. For each salinity, the SI passed through one flexible tube fitted to a peristaltic pump and was then distributed toward triplicate tanks. For each tank connected to the SI, the water flow from the SI was 11% of the total water flow (12 liters h^−1^) and was checked daily. The water input from the SI was stopped at day 6, when the recipients exhibited mortality. The other twelve tanks were not connected to the SI and were used as controls.

Survival of control and recipient oysters was followed every day for 17 days. Dead oysters were removed from tanks. Oysters were sampled at days 2 and 4 for each treatment combination, before the onset of mortality. Whole tissues from 15 oysters per tank were removed from the shells, pooled together, flash frozen and stored in liquid nitrogen.

#### Biochemical analyses and water content

Pooled oyster tissues were ground in liquid nitrogen with a MM400 homogenizer (Retsch, Eragny, France). The resulting oyster powders were stored at −80°C until further analyses. Biochemical analyses were conducted on oyster powder subsamples. Each biochemical parameter was measured at days 2 and 4 except for AMPK and fatty acids that were analyzed on day 4 only.

The water content was expressed as a percentage of water out of the total biomass. Samples of 100 mg of powder was weighed, dried at 60°C for 48 h, and weighed again.

#### Carbohydrates

Samples of 300 mg of powder were homogenized in 3 ml of nanopure water using a Polytron^®^ PT 2500 E (Kinemetica, Luzernerstrasse, Switzerland) and diluted 10 times. Carbohydrate concentrations were determined by colorimetric method according to DuBois et al. (1956). Samples (250 µl) were mixed with phenol (0.5 ml, 5% m/v) and sulfuric acid (2.5 ml, 98%), and incubated for 40 min. Absorbance was read at 490 nm with a UV 941 spectrophotometer (Kontron instruments, San Diego, CA, USA). Carbohydrates concentrations were determined using a standard calibration curve and expressed as mg of carbohydrates per g of dry weight.

#### Lipids

##### Neutral lipid class determination

Samples of 300 mg of powder were homogenized in 6 ml chloroform-methanol (2:1, v/v) according to Folch et al. (1957), then sonicated and stored at −20°C until analyses. Samples were spotted on activated silica plates using a CAMAG automatic sampler (CAMAG, Switzerland), and the plates were eluted in hexane-diethylether acetic acid (20:5:0.5 v/v/v) followed by hexane-diethylether (97:3, v/v). Lipid classes appeared as black spots after plates were dipped in a CuSO_4_–H_3_PO_4_ solution and heated. Plates were read by scanning at 370 nm, and black spots were quantified using Wincats software (CAMAG, Switzerland). This method allow separating free fatty acids, alcohols, mono-diacylglycerols, triacylglycerols (TAG), and sterols (ST). Since TAG are mainly reserve lipids and ST are structural constituents of cell membranes, the TAG-ST ratio was used as an index of the relative contribution of reserve to structure (Fraser, 1989).

##### Fatty acid composition of polar lipids

Polar lipids were extracted using a silica gel micro column (Marty et al., 1992). Aliquots of samples were evaporated until dryness and were recovered with three 500 µl washings of chloroform:methanol (98:2, v/v). Samples were then placed on the top of a silica gel micro column [30×5 mminternal diameter; Kieselgel; 70–230 mesh (Merck, Lyon, France); previously heated to 450°C and deactivated with 5% water]. The neutral lipids were removed with 10 ml of chloroform:methanol (98:2, v/v) and the polar lipids were recovered with 15 ml of methanol. A known amount of tricosaenoic acid (23:0) was added to polar fraction as an internal standard. Polar lipids were transesterified at 100°C for 10 min with 1 ml of boron trifluoride–methanol (12%Me-OH) (Metcalfe and Schmitz, 1961). This transesterification produces fatty acid methyl esters (FAME) from the fatty acid esterified at the sn-1 and sn-2 position of diacylphospholipids, and the sn-2 position of plasmalogen phospholipids. It also produces dimethyl acetals (DMA) from the alkenyl chains at the sn-1 position of plasmalogens (Morrison and Smith, 1964). Then, 1 ml of hexane and 1 ml of Milli-Q water were added, and the organic phase containing FAME and DMA was washed with 1 ml of water. FAME and DMA were analysed in a HP6890 GC system (Hewlett-Packard) equipped with a DB-Wax capillary column (30 m×0.25 mm; 0.25 μm film thickness; Agilent technologies). Peaks were analysed by comparison of their retention time with those of a standard 37 component FAME mix and other standard mixes from marine bivalves. The unsaturation index is the average number of double bonds per acyl chain. Fatty acid contents were expressed as the mole percentage of the total fatty acid content. Total DMA was used as an indicator of the plasmalogen level. The unsaturation index (UI), i.e. the sum of the mole percentage of each unsaturated fatty acid multiplied by the number of double bonds within that fatty acid, was used as an indicator of fluidity and peroxidation of membrane lipids (Hazel, 1995; Hulbert, 2003).

##### Total proteins

Proteins were extracted from oyster powder (200 mg) in ice-cold lysis buffer (2 ml), and homogenized with a Polytron^®^ PT 2500 E (Kinemetica, Luzernerstrasse, Switzerland). The buffer consisted in a mix of 150 mM NaCl, 10 mM Tris, 1 mM EDTA, 1 mM EGTA, 1% Triton X-100, 0.5% Igepal, 1 tablet of complete EDTA free protease inhibitor cocktail in 25 ml of buffer and the phosphatase inhibitor cocktail III, all at a pH 7.4 (Corporeau et al., 2012). Homogenates were centrifuged twice at 4000 rpm (3077 ***g***) for 1 h at 4°C and at 11,700 rpm (12856 ***g***) for 45 min at 4°C to eliminate the lipid fraction of the samples, using GR412 and MR22 Jouan centrifuges (Thermo electron, Massachussets, USA), respectively (Corporeau et al., 2014). The resulting supernatants were aliquoted and stored at −80°C until analyses. Protein extracts were diluted by 20× and quantified according to Lowry et al. (1951) using the DC protein assay kit (Bio-Rad, Hercules, California, USA). Absorbance was read at 540 nm and protein concentrations were determined by comparison with a calibration curve of Bovine Serum Albumin provided with the kit. Results are expressed as mg of proteins per g of dry weight.

##### SDS-PAGE and western blot

30 µg of total protein lysates were loaded on a 4-15% Criterion™ TGX Stain free™ precast polyacrymide gel (Biorad) and immunodetection was done using the rabbit polyclonal antibody AMPKα Thr172 phosphorylation (Epelboin et al., 2015). Stain-free imaging of gels and Ponceau staining of membranes were used to control for identical amounts of total protein loaded between samples.

##### Enzyme assays

All enzyme assays were performed in triplicate at room temperature. Enzyme activities were measured using Nunc™ 96-well micro-plates (Thermo Scientific), a Synergy HT micro-plate reader and the software Gen5 v2.03, both from BioTek (Winooski, Vermont, USA). Catalase activity was measured using quartz Hellma cuve 110 (Dominique Dutscher, Brumath, France) and a UV 941 spectrophotometer (Kontron instruments).

The hexokinase activity (HK; EC 2.7.1.1) was determined as described by Greenway and Storey (1999). Protein supernatant (20 µl) was mixed with assay buffer (180 µl) consisting of 100 mM Tris, 1 mM EDTA, 2 mM MgCl2, 5 mM glucose, 1 mM ATP, 0.2 mM NADP^+^ and 1 U ml^−1^ Glucose-6-Phosphate Dehydrogenase. The production of NADPH is followed by absorbance, recorded for 15 min at 340 nm. The activity was calculated using the linear portion of the kinetic (between 4 and 8 min). Results are expressed as mU mg^−1^ protein, where 1 U is the amount of enzyme necessary to oxidize 1 µmole of NADP^+^ per minute (using ε_NADH_ or _NADP+,340_=6.22 mM^−1^ cm^−1^).

The activity of citrate synthase (CS; EC 4.1.3.7), was assayed according to Childress and Somero (1979). Protein supernatant (20 µl) were added in wells with buffer (160 µl) which consisted of buffer 100 mM Tris-HCl, 0.2 mM acetyl-CoA, 0.1 mM DTNB. The reaction was initiated by addition of oxaloacetate (0.5 mM; 20 µl). Absorbance was recorded for 10 min at 412 nm. Results are expressed in mU mg^−1^ protein, where 1 U is the amount of enzyme to catalyze 1 µmole of TNB per minute (using ε_TNB,412_=13.6 mM^−1^ cm^−1^).

Total activity of superoxide dismutase (SOD; EC 1.15.1.1) was assessed using a SOD assay kit (Sigma-Aldrich, Saint-Louis, Missouri, USA). Protein supernatant (20 µl) was mixed with 200 μl of Water-Soluble Tetrazolium salt (WST-1) and with 20 μl of xanthine oxidase (XO) and xanthine mix, to initiate the reaction. After a 20 min incubation at 25°C, absorbance was read at 450 nm. The activity of SOD was determined by comparison with a standard inhibition curve performed using SOD from bovine erythrocytes. Results are expressed as in U mg^−1^ protein, where 1 U is the amount of enzyme necessary to inhibit by 50% the xanthine/xanthine oxidase complex.

The activity of catalase (CAT; EC 1.11.1.6) was assayed according to Aebi (1984). Protein supernatant (8 µl) was mixed with a 10 mM hydrogen peroxide (H_2_O_2_) solution (792 µl). Absorbance was instantaneously recorded for 90 s at 240 nm. Results are expressed as in U mg^−1^ protein, where 1 U is the amount of enzyme necessary to catalyze 1 µmole of H_2_O_2_ per minute (using ε_H2O2, 240_=0.04 mM^−1^ cm^−1^).

### Statistics

#### General linear models

Pearson correlations were calculated between salinity, proximate composition, energetic- and antioxidant-related enzyme and fatty acid composition of polar lipids. The effect of salinity on each biochemical variable was investigated by means of linear regressions. Mixed model ANOVAs were conducted to determine differences in all biochemical variables, according to salinity, infection, sampling time and their interactions. The experimental unit was the tank in which salinity and infection were applied. Main plots were salinity and infection levels, subplots were sampling times. For models with statistical significance (*P*<0.05), Tukey multiple comparison tests (*P*<0.05) were used to determine differences among treatments. The general linear models were conducted using the R Software version 3.3.3 (www.R-project.org).

#### Cox model

To investigate how does oyster metabolism influence the risk of disease induced mortality, the relationship between the mortality risk and the metabolic parameters (energetic reserves, key enzyme activities and fatty acid levels) were examined using Cox proportional hazards regression models (Cox, 1972). The survival of control oysters (not exposed to the source of infection) was not included in the statistical model because it was always >95% (Fuhrmann et al., 2016). Adjusted risks were determined by including salinity. Because the proportionality of hazards assumption was violated, time-dependent covariates representing the interaction of the salinity and time were added in the adjusted model. Significant explanatory covariates (*P*<0.05) were selected using a stepwise method and tested in the multivariate Cox regression model. These analyses were conducted using the SAS software package (SAS 9.4, SAS Institute).

## Supplementary Material

Supplementary information
