# Supplementary material for: Metabolism of the Pacific oyster, Crassostrea gigas, is influenced by salinity and modulates survival to the Ostreid herpesvirus OsHV-1
Source: Biol Open. 2018 Feb 15;7(2):bio028134. doi: 10.1242/bio.028134 (PMC5861354; doi:10.1242/bio.028134)
Supplement: Supplementary information [file biolopen-7-028134-s1.pdf]

Table S1: Proximate composition of oysters as a function of salinity (‰), infection (controls vs. recipients) and time (day 2 vs 4). Values are means ± SE between replicate tanks (n=3). Abbreviations: DW, Dry Weight; TAG/ST, ratio Triglyceride/Sterol

Variables	Time	Controls				Recipients			
		10	15	25	35	10	15	25	35
Water (%)	2	88.9 ± 0.3	87.8 ± 0.6	87.0 ± 0.1	87.5 ± 0.6	88.9 ± 0.3	88.5 ± 0.3	86.8 ± 0.4	85.7 ± 0.2
	4	87.5 ± 0.2	86.5 ± 0.4	86.3 ± 0.2	86.1 ± 0.4	88.3 ± 0.3	88.0 ± 0.26	86.6 ± 0.1	85.6 ± 0.2
Protein (mg g^{-1} DW)	2	450.0 ± 27.4	470.8 ± 9.5	416.2 ± 5.7	403.0 ± 19.6	484.4 ± 13.2	480.4 ± 12.3	406.2 ± 13.5	375.5 ± 2.9
	4	444.5 ± 28.9	413.2 ± 7.6	387.4 ± 14.5	380.2 ± 10.5	488.7 ± 10.6	444.6 ± 26.3	409.6 ± 16.3	370.2 ± 22.6
Carbohydrate (mg g^{-1} DW)	2	191.6 ± 32.1	176.7 ± 7.0	111.7 ± 9.7	108.8 ± 9.1	164.7 ± 17.5	131.2 ± 4.4	114.0 ± 9.0	108.8 ± 7.9
	4	209.3 ± 12.9	168.9 ± 1.5	113.0 ± 1.9	98.5 ± 0.4	173.3 ± 8.4	176.4 ± 16.2	132.1 ± 3.7	118.7 ± 11.5
TAG/ST	2	4.3 ± 0.2	3.9 ± 0.2	3.3 ± 0.3	2.9 ± 0.2	4.5 ± 0.1	3.5 ± 0.3	4.2 ± 0.2	4.1 ± 0.2
	4	4.4 ± 0.1	3.9 ± 0.1	4.4 ± 0.2	3.6 ± 0.4	4.6 ± 0.5	3.7 ± 0.1	4.7 ± 0.2	4.1 ± 0.6

Table S2: Results of the split-plot ANOVA investigating the effect of salinity, infection and time and their interactions on proximate composition of oysters. Significant p-values (p < 0.05) are in **bold.**

		Water		Protein		Carbohydrate		TAG/ST	
Source of variation	d.f	F	p	F	p	F	p	F	p
Mainplot									
Salinity	3	49.00	< **0.001**	19.01	< **0.001**	27.47	< **0.001**	4.88	**0.014**
Infection	1	0.37	0.552	1.68	0.213	1.14	0.301	4.42	0.052
Salinity × Infection	3	11.15	< **0.001**	1.81	0.187	2.33	0.113	2.62	0.087
Error a: Tank × Salinity × Ifection	16								
Subplot									
Time	1	18.47	**0.001**	5.74	**0.029**	4.09	0.060	8.33	**0.011**
Salinity × Time	3	0.43	0.736	1.63	0.222	0.60	0.623	2.04	0.149
Infection × Time	1	5.66	**0.030**	1.73	0.207	3.91	0.066	2.16	0.161
Salinity × Infection × Time	3	0.26	0.852	0.09	0.964	1.55	0.240	0.74	0.543
Error b: Tank × Salinity × Infection × Time	16								

Biology Open • Supplementary information

Table S3: Activities of energetic and antioxidant related enzymes as a function of salinity (%), infection (controls vs. recipients) and time (day 2 vs 4). Values are means ± SE between replicate tanks (n=3). Abbreviations: CS, Citrate Synthase; HK, Hexokinase; SOD, Superoxyde dismutase; CAT, Catalase.

Variables	Time	Controls				Recipients			
		10	15	25	35	10	15	25	35
HK	2	1.2 ± 0.2	1.4 ± 0.1	1.9 ± 0.1	2.1 ± 0.2	1.5 ± 0.1	1.3 ± 0.1	2.0 ± 0.1	2.5 ± 0.1
(mU mg^{-1})	4	1.5 ± 0.2	1.8 ± 0.1	2.1 ± 0.1	1.8 ± 0.1	1.7 ± 0.2	1.7 ± 0.1	1.9 ± 0.1	2.3 ± 0.1
CS	2	23.8 ± 1.6	28.2 ± 0.7	25.0 ± 1.1	24.6 ± 1.5	25.8 ± 1.3	22.0 ± 1.7	26.8 ± 4.4	24.0 ± 0.2
(mU mg^{-1})	4	28.5 ± 3.6	30.5 ± 3.0	30.1 ± 3.0	22.7 ± 0.1	33.2 ± 0.5	27.7 ± 1.3	31.5 ± 1.3	28.4 ± 1.7
SOD	2	11.8 ± 3.1	9.9 ± 0.4	11.5 ± 2.0	12.7 ± 1.8	9.8 ± 1.6	12.6 ± 1.9	12.3 ± 1.8	9.8 ± 1.3
(U mg^{-1})	4	5.2 ± 0.7	5.7 ± 0.4	4.3 ± 0.7	5.5 ± 0.5	4.5 ± 0.4	6.0 ± 0.6	5.7 ± 0.2	4.5 ± 1.0
CAT	2	32.5 ± 5.1	38.2 ± 2.3	40.8 ± 1.3	42.0 ± 3.9	22.8 ± 1.0	36.8 ± 1.8	38.6 ± 0.9	33.9 ± 1.0
(U mg^{-1})	4	31.6 ± 2.6	41.0 ± 3.3	50.8 ± 4.0	44.3 ± 2.5	26.8 ± 1.9	29.9 ± 1.1	43.4 ± 3.3	40.8 ± 3.4

Biology Open • Supplementary information

Table S4: Results of the split-plot ANOVA investigating the effect of salinity, infection and time and their interactions on enzyme activities in oysters. Significant p-values (p < 0.05) are in **bold**. Abbreviations: CS, Citrate Synthase; HK, Hexokinase; SOD, Superoxyde dismutase; CAT, Catalase.

		HK		CS		SOD		CAT	
Source of variation	d.f	F	p	F	p	F	p	F	p
Mainplot									
Salinity	3	46.45	< **0.001**	1.71	0.206	0.17	0.913	22.68	< **0.001**
Infection	1	11.16	**0.004**	0.47	0.503	0.06	0.806	19.73	< **0.001**
Salinity × Infection	3	6.30	**0.005**	2.37	0.108	1.22	0.335	0.13	0.939
Error a: Tank × Salinity × Infection	16								
Subplot									
Time	1	2.36	0.144	20.45	< **0.001**	106.32	< **0.001**	4.32	0.054
Salinity × Time	3	3.36	**0.045**	1.32	0.304	0.28	0.840	2.17	0.132
Infection × Time	1	0.26	0.616	2.70	0.120	0.11	0.743	0.24	0.631
Salinity × Infection × Time	3	0.34	0.795	0.58	0.636	0.71	0.558	1.77	0.193
Error b: Tank × Salinity × Infection × Time	16								

Table S5: Results of the ANOVA investigating the effect of salinity, infection and time and their interactions on the fatty acids composition of the polar lipids of oysters. Only fatty acids contributing to >1% were considered. Significant p-values (p < 0.05) are in **bold.**

Source of variation		14:0		16:0		16:1n-7		18DMA		18:0		18:1n-9	
	d.f	F	p	F	p	F	p	F	p	F	p	F	p
Salinity	3	10.44	< **0.001**	1.23	0.333	3.14	0.055	4.98	**0.013**	18.87	< **0.001**	22.43	< **0.001**
Infection	1	29.25	< **0.001**	1.57	0.228	1.90	0.188	0.69	0.418	1.76	0.203	4.22	0.057
Salinity × Infection	3	4.20	**0.023**	1.09	0.383	2.15	0.134	0.67	0.581	1.73	0.201	0.67	0.583
Error : Tank × Salinity × Infection	16												
Source of variation		18:1n-7		18:2n-6		18:3n-3		18:4n-3		20:1 dma		20:1n-11	
	d.f	F	p	F	p	F	p	F	p	F	p	F	p
Salinity	3	83.61	< **0.001**	10.88	< **0.001**	4.03	**0.026**	11.95	< **0.001**	11.17	< **0.001**	13.79	< **0.001**
Infection	1	8.96	**0.009**	9.84	**0.006**	17.36	**0.001**	36.44	< **0.001**	1.05	0.320	0.08	0.786
Salinity × Infection	3	2.16	0.133	1.14	0.362	2.08	0.143	2.62	0.087	0.21	0.890	1.62	0.225
Error : Tank × Salinity × Infection	16												
Source of variation		20:1n-7		20:4n-6		20:5n-3		22:5n-6		22:5n-3		22:6n-3	
	d.f	F	p	F	p	F	p	F	p	F	p	F	p
Salinity	3	2.77	0.076	171.48	< **0.001**	27.69	< **0.001**	79.69	< **0.001**	35.83	< **0.001**	9.42	< **0.001**
Infection	1	0.58	0.459	8.92	**0.009**	0.40	0.537	3.40	0.084	0.49	0.494	0.93	0.350
Salinity × Infection	3	0.63	0.607	0.38	0.769	0.46	0.713	1.06	0.395	0.54	0.659	1.28	0.315
Error : Tank × Salinity × Infection	16												
Source of variation		20NMI		22NMI		Branched FA		Unsaturation index		∑n6		∑n3	
	d.f	F	p	F	p	F	p	F	p	F	p	F	p
Salinity	3	3.83	**0.030**	11.61	< **0.001**	7.39	**0.003**	9.34	< **0.001**	120.54	< **0.001**	7.76	**0.002**
Infection	1	0.07	0.793	1.10	0.310	2.54	0.131	0.16	0.691	0.22	0.645	8.45	0.010
Salinity × Infection	3	2.98	0.063	0.74	0.546	3.37	0.045	2.37	0.109	0.54	0.662	0.79	0.515
Error : Tank × Salinity × Infection	16												
Source of variation		∑n3/n6											
	d.f	F	p										
Salinity	3	140.29	< **0.001**										
Infection	1	4.14	0.059										
Salinity × Infection	3	0.44	0.728										
Error : Tank × Salinity × Infection	16												

Table S6: Association between metabolic parameters (energetic reserves, key enzyme activities and fatty acid levels) and the risk of death of oysters exposed to OsHV-1 by a Cox proportional hazards model. For each covariate, the following elements are provided: its parameter estimate and its standard error (SE), the χ^2 statistic (with 1 degree of freedom for all tests), the resulting p value for the type II test and the corresponding instantaneous odds ratio. Only significant covariates are presented.

Covariate	Estimate	SE	χ^2	p value	Odds ratio
∑n-3/∑n-6 d4	-3.885	0.454	73.4	<0.0001	0.02
∑n-6 d4	0.694	0.089	61.0	<0.0001	2.00
22:5n-6 d4	3.468	0.506	47.0	<0.0001	32.08
20:2NMI d4	-6.901	1.192	33.5	<0.0001	0.00
AMPK d4	-0.029	0.005	32.9	<0.0001	0.97
Carbohydrate d2	-0.032	0.006	31.2	<0.0001	0.97
20:4n-6 d4	0.635	0.118	28.9	<0.0001	1.89
22:5n-3 d4	-6.986	1.362	26.3	<0.0001	0.00
CAT d4	0.087	0.017	25.9	<0.0001	1.09
CAT d2	0.141	0.028	24.6	<0.0001	1.15
∑n-3 d4	-1.335	0.277	23.3	<0.0001	0.26
16:1n-7 d4	3.979	0.892	19.9	<0.0001	53.44
Carbohydrate d4	-0.018	0.005	16.6	<0.0001	0.98
20:5n-3 d4	-1.490	0.368	16.4	<0.0001	0.23
18:1n-9 d4	-4.400	1.091	16.3	<0.0001	0.01
Proteins d2	-0.014	0.004	15.4	<0.0001	0.99
22:2NMI d4	-1.660	0.466	12.7	0.0004	0.19
Water content d4	-0.555	0.158	12.4	0.0004	0.57
18:1n-7 d4	0.849	0.246	11.9	0.0006	2.34
Proteins d4	-0.011	0.003	11.7	0.0006	0.99
HK d2	1.060	0.324	10.7	0.0011	2.89
Water content d2	-0.444	0.139	10.3	0.0014	0.64
Branched FA d4	-16.196	5.091	10.1	0.0015	0.00
18:0 d4	1.642	0.543	9.2	0.0025	5.17
Unsaturation index d4	0.204	0.068	9.1	0.0025	1.23
14:0 d4	-1.942	0.660	8.7	0.0032	0.14
18:2n-6 d4	-2.230	0.770	8.4	0.0038	0.11
20:1DMA d4	-1.233	0.448	7.6	0.0059	0.29
22:6n-3 d4	1.137	0.476	5.7	0.0169	3.12
HK d4	1.265	0.537	5.5	0.0186	3.54
18:0DMA d4	-0.546	0.233	5.5	0.0190	0.58
16:0 d4	-2.258	0.985	5.3	0.0219	0.11
20:1n-7 d4	1.822	0.847	4.6	0.0314	6.19
20:1n-11 d4	-2.551	1.278	4.0	0.0459	0.08

The tested covariates were water content, protein, carbohydrate, TAG/ST, CS, CAT, SOD, HK and PK measured at day 2 and 4. Also, the main fatty acids, namely 14:0, 16:0, 16:1n-7, 18:0DMA, 18:0, 18:1n-9, 18:1n-7, 18:2n-6, 18:3n-3, 18:4n-3, 20:1DMA, 20:1n-11, 20:1n-7, 20:4n-6, 20:5n-3, 22:5n-6, 22:5n-3, 22:6n-3, 20:2NMI, 22:2NMI, branched FA, unsaturation index, ∑n-6, ∑n-3, and the ratio ∑n-3/∑n-6 at day 4 were included. Abbreviations: d, day.

Biology Open • Supplementary information
